# Vein Pattern Locating Technology for Cannulation: A Review of the Low-Cost Vein Finder Prototypes Utilizing near Infrared (NIR) Light to Improve Peripheral Subcutaneous Vein Selection for Phlebotomy

**DOI:** 10.3390/s19163573

**Published:** 2019-08-16

**Authors:** Cheng-Tang Pan, Mark D. Francisco, Chung-Kun Yen, Shao-Yu Wang, Yow-Ling Shiue

**Affiliations:** 1 Department of Mechanical and Electro-Mechanical Engineering, National Sun Yat-sen University (NSYSU), Kaohsiung 80424, Taiwan; 2 Institute of Medical Science and Technology, NSYSU, Kaohsiung 80424, Taiwan; 3 Institute of Biomedical Sciences, NSYSU, Kaohsiung 80424, Taiwan; 4 College of Medical Technology, Trinity University of Asia (TUA), Quezon City 1102, Philippines

**Keywords:** (IR) infrared, (LED) light emitting diode, vein finder, cannulation, venipuncture, phlebotomy, hemoglobin

## Abstract

One of the most common means for diagnosis is through medical laboratory testing, which primarily uses venous blood as a sample. This requires an invasive method by cannulation that needs proper vein selection. The use of a vein finder would help the phlebotomist to easily locate the vein, preventing possible pre-analytical error in the specimen collection and even more discomfort and pain to the patient. This paper is a review of the scientific publications on the different developed low-cost vein finder prototypes utilizing camera assisted near infrared (NIR) light technology. **Methods**: Electronic databases were searched online, these included PubMed (PMC), MEDLINE, Science Direct, ResearchGate, and Institute of Electrical and Electronics Engineers (IEEE) Xplore digital library. Specifically, publications with the terms vein finder prototype, NIR technology, vein detection, and infrared imaging were screened. In addition, reference lists were used to further review related publications. **Results**: Cannulation challenges medical practitioners because of the different factors that can be reduced by the utilization of a vein finder. A limited number of publications regarding the assessment of personnel performing cannulation were observed. Moreover, variations in methodology, number of patients, type of patients according to their demographics and materials used in the assessment of the developed prototypes were noted. Some studies were limited with regard to the actual human testing of the prototype. **Conclusions**: The development of a low-cost effective near infrared (NIR) vein finder remains in the phase of improvement. Since, it is being challenged by different human factors, increasing the number of parameters and participants/human for actual testing of the prototypes must also be taken into consideration for possible commercialization. Finally, it was noted that publications regarding the assessment of the performance of phlebotomists using vein finders were limited.

## 1. Introduction

Medical laboratory diagnostics is an important part of patient care by personalized (precision) medicine. It must be with advancement in order to achieve a greater degree in quality healthcare, especially from activities starting with ordering, obtaining and handling of biological specimens [1]. This includes the blood sample as a common biological specimen. The said processes are part of the pre-analytical phase [2]. In previous studies, pre-analytical errors were the most commonly reported with the highest percentage of errors in the clinical laboratory, which include specimen collection by phlebotomy also known as venipuncture. With these challenges, the integrity of the biological samples that should represent the biological status in vivo was affected leading to unreliable diagnostic information [3].

A study showed that about 90% of hospitalized patients may require peripheral cannulation for the intravenous route of treatment and more than one billion venipunctures per year are being performed as a basic requirement for most diagnostic tests [4]. Venipuncture refers to the practice of drawing blood by penetrating the vein’s wall with a needle for collection. It is considered as an invasive procedure which can cause patient’s distress, pain and even unavoidable extreme reactions from children, adults or patients with mental illness [5] and frustration to the clinical laboratory scientist/phlebotomist and even to the attending physician due to unsuccessful procedure.

Moreover, failure in venipuncture can cause thrombosis of the vein [4], hematoma, or even nerve injury involving the lateral antebrachial cutaneous nerve (LACN), which can lead to the so-called “causalgia” or complex regional pain syndrome (CRPS) [5,6].

Tourniquet application and the process of slightly tapping the site for venipuncture, commonly the antecubital fossa, can be applied to stimulate the vein and to be able to locate it [5]. Locating the vein is not easy; there are some factors that affect patients’ veins visibility such as variation on the skin tone of the young children (especially infants), obesity and dehydrated patients [7].

Recently, a technological development provided trans-illuminating devices, which uses the near infrared light-emitting diodes (NIR-LED) to visualize superficial veins with the hemoglobin absorbing of the light emitted, forming an image on the skin surface [5]. Hemoglobin is a heterotetramer composed of subunits of alpha- and beta-globin (the protein part) that is bound to a heme prosthetic group (iron containing compound) [8]. As to its major function, it is able to transport oxygen (O2) rich blood (oxyhemoglobin) from the lungs to the peripheral tissues and carbon dioxide (CO2) rich blood (deoxyhemoglobin) with the opposite route [9].

The imaging, through the use of trans-illuminating devices, allows a deeper look below the skin with best wavelength range for better contrast between skin and veins image [7].

The review emphasizes the strengths and weaknesses of previous studies on vein finder prototype. This may serve as a guide for future researches in the development of a low-cost effective vein finder for medical application and possible commercialization. Furthermore, this technology will open opportunities for collaborative studies between medical practitioners and professionals in the field of medical engineering.

## 2. Methods

**Figure 1:**
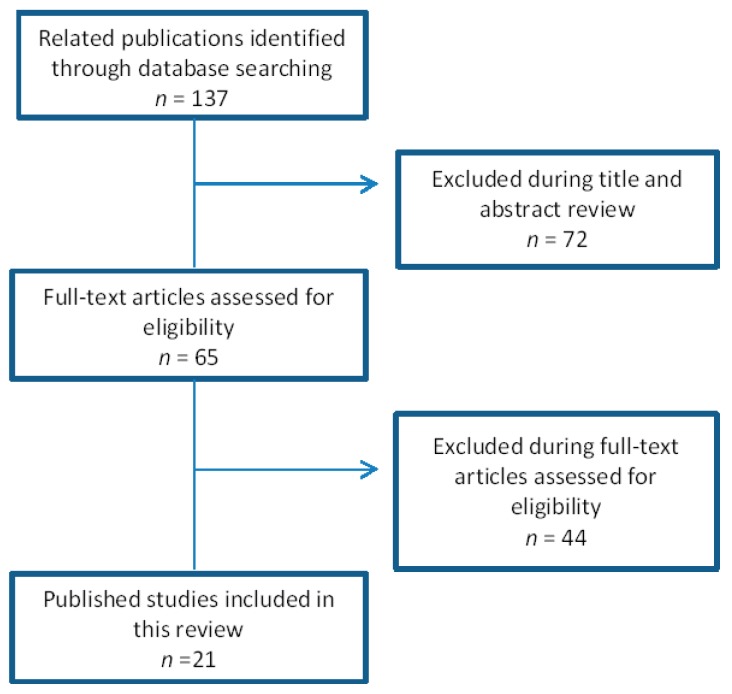
Research strategy on digital library databases.

This article, reviews scientific publications regarding the different developed near infrared light emitting diode (NIR-LED) vein finder prototypes using low cost materials. It focuses on the principle, materials, parameters and evaluation done with the prototype. The data analysis included studies written in English published from 2007 to 2018. Electronic databases were searched online, PubMed, MEDLINE, Science Direct, ResearchGate, and the most informative site for the subject matter, The Institute of Electrical and Electronics Engineers (IEEE) Xplore digital library database. Specifically, publications with the terms vein finder prototype, near infrared (NIR) technology, vein detection, and infrared imaging were screened. Related publications were identified through database searching after the preliminary evaluation based on the specification needed in this review paper. Exclusion started with the title and abstract review, followed by full-text articles assessment for eligibility with emphasis to studies directly associated with vein finder prototypes using NIR technology. In addition, reference lists were used to review related publications (see Figure 1). The research strategy on digital library databases is shown below.

## 3. Results and Analysis

### 3.1. Errors in the Pre-Analytical Phase in Clinical Laboratory Testing

Cannulation using the vein is the most common site for blood extraction, also known as phlebotomy, gaining venous blood as a specimen of choice for many clinical laboratory testing for the diagnosis of diseases. Moreover, cannulation is being used in performing technical procedures in emergency medicine [10]. It includes peripheral intravenous catherization (PIVC) that remains challenging for many clinicians to complete successfully on the first attempt [11]. This procedure is considered as one of the pre-analytical steps that can be a possible source of variability in the laboratory results affecting proper diagnosis and treatment process (see Table 1). Table 1 shows that pre-analytical phase in clinical laboratory testing in various studies has the highest percentage error as compared to the analytical and post analytical phases from 46.00% up to 77.10% with the average of 61.55%.

In venipuncture, there are means on how to help the phlebotomist view the vein properly, among those were by slightly tapping the site [5], use of tourniquet, and applying cold vibration close to the site for venipuncture or fist clenching, which are considered and studied to cause physiological disturbance on veins and significant variability in several laboratory test results [19].

The procedure for venipuncture cannot be considered as a gold standard yet, since errors are still being detected [20] with continuous recommendations for improvements were supplemented. The CLSI H03-A6 document which suggested modifications in some of the steps in venipuncture, such as to cleanse the site and to allow drying before applying tourniquet to view and select the vein and finally to release the tourniquet immediately when the filling of the first tube started. It is able to reduce the tourniquet application time, as the result showed in the study by [20] Oliveira G. et al., in 2013. Moreover, it was mentioned that the transilluminator (vein finder) can make for more accurate visualization of the vein course, unfortunately, it is not widely used mainly due to the cost of the commercially available devices [19].

The patient’s vein is the main site for blood specimen collection for testing, entry point for medications, and the site for and blood transfusions. Venipuncture should be performed with care and precise considering the limited number of easily accessible veins in the body [21].

Aside from venipuncture, the near infrared (NIR) transilluminator imaging was used to enhance vein treatment by helping in the identification of veins which were noted to be invisible to the naked eye but too shallow for ultrasound detection [22]. It is even used for optical imaging of the skin to guide dermatologist for proper diagnosis of diseases and skin care, specifically in the study by [23] Huang K et al., 2012, shows black/white mole skin roughness imaging in epidermis and skin tissue aging or skin lesions.

Cannulation is being considered as challenging due to many factors. To mention, the following are the known risk factors that cause difficulties during this procedure: Dehydration, dark skin, obesity, hypotension, peripheral vasoconstriction, poor vein quality, aging, young age including preterm and term neonates, vein site used in drug abuse and chemotherapy, telangiectasia, site with skin rash, including even the low skill of the personnel performing the procedure [24].

These challenges necessitate for the improvement of steps in performing cannulation. It includes development and use of a vein finder to assist in locating vein as the site for different procedures, which aim to lessen missed vein during needle insertion on the first trial by medical practitioners.

### 3.2. Studies Regarding the Assessment on Vein Visualization by Conventional or Standard Method (Visualization or Palpation) and with the Use of Infrared Vein Finder Device

A limited number of publications regarding the assessment for personnel performing venipuncture was observed and variations in the methodology, number of patients, type of patients according to their demographics and materials used in developing a vein finder prototype were noted. The success in the cannulation process does not merely rely on the vein finder but more so to the skills of the medical practitioner.

The Chiao et al., 2013 [25] study showed that the possible cannulation site with the use of a vein finder device has better results with 9.1 (95% CI 8.6–9.5) compared with the conventional method 5.8 (95% CI 5.4–6.2). In addition, the result shows that African-American or Asian ethnicity, and obesity were associated with decreased vein visibility, and these were also been observed in the Sebbane et al., 2012 [10] study with consideration on obesity as an independent risk factor for difficult peripheral venous access.

While in 2012 Phipps et al., [26] a study based on successful first attempt for peripherally inserted central catheter shows 59.00% (33/56) for standard technique (visualization or palpation) and 64.00% (38/59) with a vein finder. Recently, a 2016 study by Barreras and Chang [27] revealed that the use of a near infrared (NIR) device significantly increased intravenous access success rates compared with visualization or palpation 26% vs. 19.6%, respectively in children with special health care needs.

Moreover, the Fukuroku et al., 2016 [28] study on validating the effectiveness of vein infrared visualization performed by second-year nursing students, confirmed that the vein viewer contribute immensely to the improvement of their practical skills in venipuncture, specifically, in the site selection time with the context for the elderly patients.

### 3.3. Basic Composition, Its Principle and Designs of a Vein Finder Prototype

**Figure 2:**
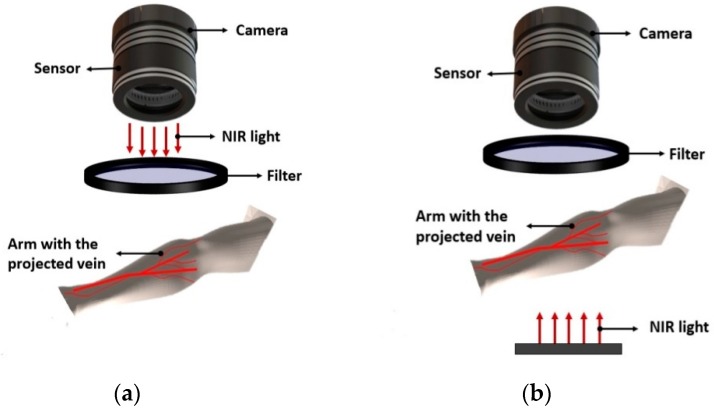
Basic components of a near infrared (NIR) vein finder prototype: (**a**) Reflected light type, and (**b**) transillumination type.

A basic prototype vein finder is composed of a high power NIR-LED as the light source, a compact infrared-sensitive camera, sensor that will capture and format the image and a filter to remove unwanted impurities [29,30,31,32,33,34] (see Figure 2). Other prototypes were incorporated with additional parts by the researchers for enhancement purposes, like the use of a Digital Light Processing (DLP) projector that allows vein image on the surface of the skin [35].

**Figure 3:**
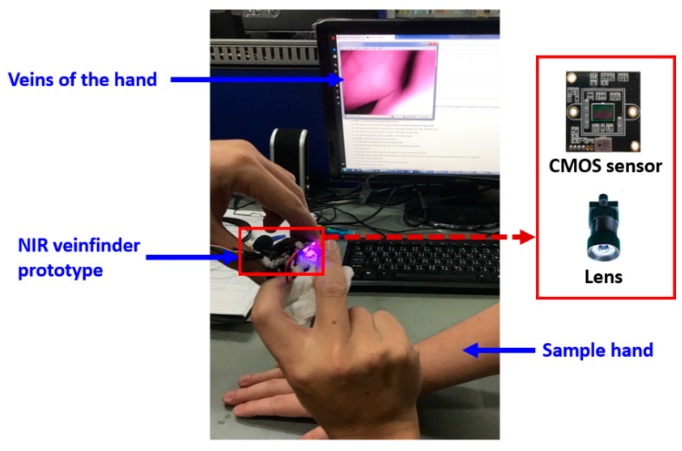
Sample projected vein image of the dorsal hand using NIR vein finder prototype.

The principle of a vein finder device is based on two illumination types: Reflected light and transillumination. For reflected light, the light from the source was reflected at the site, showing an example of the hand surface and then captured by the camera; this principle was usually applied by a commercial hand-vein scanner. While for transillumination, the light penetrates the skin and tissue of the site and then the image will be captured by the camera [34]. The image in transillumination is due to the presence of deoxyhemoglobin in venous blood which absorbs the red light and illuminates the veins as dark lines on the skin surface, allowing the phlebotomist/medical practitioners to locate the vein [36,37]. This is composed of two stages, firstly; the infrared (IR) light penetrates the human tissue and secondly, deoxyhemoglobin in the venous blood absorbs more of the incident IR light than the surrounding tissue [31]. The reflected light type of a vein finder shown in Figure 2a became more popular in commercial devices due to its characteristics, such as: It requires lower light intensity; less power consumption suitable for battery operated devices, and enables a compact design vein finder. While transillumination devices is the opposite, which require a higher light intensity that uses more power and non-compact with the arm or hand being scanned were placed in between the light source and the camera shown in Figure 2b [34] (see Figure 3). The sample projected a vein image of the dorsal hand using the NIR vein finder prototype.

Recently, the use of the near infrared region (NIR) of the electromagnetic spectrum became more popular in developing a low cost but efficient type of vein finder. This is to primarily address the estimated cost of available commercial vein finder using NIR technology for about 4500 USD (portable) to 27,000 USD (non-portable) [38]. Moreover, the most significant reason is the challenges that the physician, nurse, emergency medical technician and other medical practitioners encountered during venipuncture [37], intravenous (IV) drug delivery [32] and cannulation. This is usually due to the following reasons; veins are either very small, and/or deep, the patient’s age (elderly or pediatrics), skin color [37], and obesity level [32,35]. It often results in multiple attempts in needle insertion leading to pain, discomfort, dissatisfaction, delayed treatment [37], hematoma, swell in the skin, cuts on the bone [39], bleeding, and even infection. Especially in young children, despite the dedicated and highly skilled physicians, multiple attempts are often unavoidable, which can be traumatic [40], even requiring blind puncture and may sometimes needs general anesthesia [29]. In fact, studies reported that in consideration of the factors stated above, it leads to a significantly low first-attempt successful rate in venipuncture [41].

### 3.4. Characteristics of the Different Vein Finder Prototypes

Table 2 shows the different prototypes with their basic characteristics, including the parameters considered by the researchers for the purpose of their evaluation.

## 4. Discussion

Characteristics of the different vein finder prototypes based on parameters used and evaluation process are documented below.

### 4.1. Wavelength

In the electromagnetic spectrum’s range of 740 nm to 940 nm, the light can penetrate to about 5 mm deep of the skin tissue reaching the deoxygenated hemoglobin. The vein with the deoxygenated hemoglobin forms a dark contrast to the skin tissue due to the higher light absorption coefficient and lower backscatter light coefficient [48].

**Figure 4:**
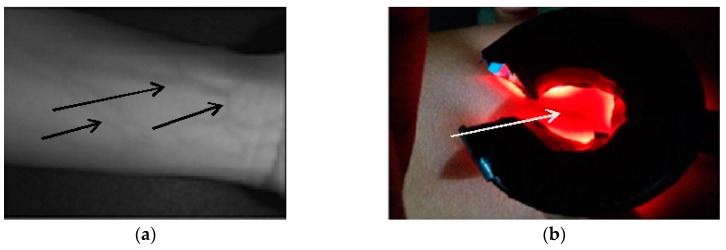
Sample veins image from different wavelengths: (**a**) 850 nm [50] and (**b**) 696 nm [43].

Based on the studies presented, there were variations in wavelength set by different researchers, 9 out of 21 (42.85%) used 850 nm, followed by 4 out of 21 (19.04%) for both 940 nm and 740 nm, and the remaining 19.07% are the studies with varying wavelength. From Table 2, Dhakshayani, M. et al., 2015 [47] showed that optimizing the wavelength by utilizing a multispectral IR source, can achieve good visibility of veins considering the types of patients based on color, age and tissue thickness, also the near infrared rays of shorter wavelengths 740, 765 nm exhibits high absorption of light by deoxygenated hemoglobin, with deep penetration at a longer wavelength of 770 and 780 nm. A similar study by Wang F et al., 2013 [50] stated that using multispectral imaging IR with a significant finding that a hairy forearm affects the image of the vein by the formation of a strong reflectance glare while, Kimori et al., 2015 [46] used 850 nm transmitted through the subcutaneous tissue and being compared as deeper than the light source of a commercial vein finder. Another study showed variation in the selected wavelength based on skin color. For fair skin, the image quality was reported to be slightly better in the range of 750 to 800 nm as compared to darker skin having the overall range considered suitable is 800 to 850 nm [7]. Among the studies, Anupongongarch et al., 2015 [37] used the light source with the highest wavelength up to 1000 nm. This variation indicates that the standardization for the optimum wavelength for the purpose of vein viewing is not been set to date yet. See Figure 4, sample vein images at a given commonly used wavelength of 850 nm and with a lower wavelength of 696 nm.

### 4.2. Types of Camera, Sensors: Charge-Coupled Device (CCD) and Complementary Metal Oxide Semiconductor (CMOS) and Filters

As to the type of camera with its sensor, 6 out of 21 (28.57%) studies presented used the CCD, and 5 (23.80%) with CMOS. To mention there were 5 (23.80%) which did not mention the type of utilized sensor, while there is a study that used both types of sensor. The use of a simple camera such as the camera phone in 3 out of 21 (14.28%) and the webcam in 1 out of 21 (4.76%) were also considered. Finally, among the studies, there were 2 (9.52%) which did not include a camera in their prototypes. See Table 2.

A sensor has the capability to detect the reflected light and capture images real time [49]. There are two commonly used sensors; the CCD and the CMOS. The charge-coupled device CCD sensors were used in cameras that were known to create high-quality, low-noise images with lots of pixels and excellent light sensitivity, while CMOS were noted to be low as compared to performance, but traditionally, consume less power and are inexpensive so that, nowadays, they are being improved [54].

CMOS image sensors are replacing CCD in many applications, with characteristics related to the possibility to directly interface the photosensitive device with electronic read-out at the pixel level and with better on-chip functionality [55]. Recently, with such applications like motion detection, mobile-phones, wearable devices and especially in bio-medical applications such as in-vivo imaging sensors [56], a sensor device that enables the multi-functional recording of brain activities such as intrinsic optical signals (IOS), electroencephalogram (EEG), imaging and electrical stimulation that can be utilized for diagnosis during surgical procedure [57]. With this, studies were noted using CMOS, Dhakshayani M. and Yacin S, 2015 [47] stating that CMOS based sensors as IR detector has high uniformity, low noise, low power consumption and with a highspeed performance/faster readout. Another study by Marathe M. et al., 2014 [31] used CMOS over the CCD, due to cost and availability.

Optical filters and diffusers were incorporated in the device in order to block wavelengths outside of the near-infrared range and to spread the light evenly on the skin of the target site, respectively. Simple materials such as: A plastic sheet of the floppy disks or negative films, as an inexpensive IR filter that can block wavelengths less than 600 nm and tissue paper and frosted window films, as a diffuser of light from light emitting diodes (LEDs) can be used for the said purpose [42]. Other materials used such as a common filter were the following: Kodak wratten 87 IR filter [47], pass-through filter (exposed and developed empty 35 mm camera film) [58], butter paper [52], polarizing filters and blank sheet made of polycarbonate noted with a very high absorption rate at a given wavelength [53]. While, the Wang F. et al., 2013 [50] prototype has a multi-filter, a 546 and 570 nm narrow-band filters for the de-oxy and oxy forms of hemoglobin, respectively, a 475 nm broadband filter for the strong absorption bands of pigmented substances such as melanin, beta-carotene, and hemoglobin, a 615 nm filter for a region of little absorbance by skin pigments, and 850 nm filter for the NIR band that includes absorption by lipids but excludes absorption by water.

Also, it was noted that digital single lens reflex (DSLR) camera gives better results for the image captured compared to poor results for images obtained with the use of web-camera that can be attributed to the low contrast quality. As to the time-of-flight (TOF) camera, it provides fast acquisition of Cartesian coordinates for enabling the localization of the target veins [44].

In the study by Ayoub Y et al., in 2018 [33], contrast-limited adaptive histogram equalization (CLAHE) was utilized for the purpose of enhancing the image contrast. It provides a clearer vein image for visualization. CLAHE was developed in 1994, an algorithm which cut the produced histogram of the dark level of a picture then redistributed the pixels over the entire histogram, resulting into an enhanced image [59].

For the ordinary camera phones with Video Graphics Array (VGA) quality pictures, it has a capability to detect infrared images, but optimizing the quality of a mobile camera with higher resolutions are preferred. In addition, the system for auto white balance must be used for optimum viewing [52].

The role of a compact infrared-sensitive camera in a vein finder is important in giving a clearer image of the light reflected or transilluminated by hemoglobin present in the blood. This image is the result of the reaction of hemoglobin in the blood with light that makes the vein looks darker against the skin surface [43].

With these variations in the materials being utilized for the development of a vein finder, such as the types of camera, sensors, and filters, it shows that we are still searching for the best components of a vein finder specifically useful for medical use. Currently, this technology is commercially available but very expensive. To answer this concern, we may further study this technology, to improve the required specifications for the development of a low-cost effective vein finder.

### 4.3. The Common Parameters Used in the Assessment of the Prototypes Were the Following: Body Mass Index (BMI), Skin Color/Tone, Age, and Site for Venipuncture

Any device prior to commercial use should be thoroughly tested and evaluated. This requires the consideration of parameters that will measure full function of the device. For the vein finder prototype, among the studies reviewed the following common parameters were used: Body mass index (BMI)—5 out of 21 (23.80%), skin color/tone 8 (38.09%), age 5 (23.80%), gender 3 (14.28%), specified site for testing 8 (38.09%), and there were 8 (38.09%) of the studies, which did not include any parameters in their testing (see Table 2). Statistics shows that there was a significant number, 8 out of 21 (38.09%) of studies, without inclusion of parameters as part of the assessment process, which limits the chance to measure the full capability of the prototype.

On the other hand, there were some additional parameters included by other researchers in their studies such as body temperature, hemoglobin, race (Asian and Caucasian), fat padding and hairy forearm. The use of different parameters for the assessment of the vein finder prototype is very significant, since it can be considered as variables that may affect the capability of the vein finder to locate the peripheral subcutaneous vein.

#### 4.3.1. Body Mass Index (BMI)

The BMI is an estimate of body fat based on height and weight for calculation that applies to both adult men and women. BMI is a useful measure for indicating the characteristic of being overweight and/or obesity. The classification of BMI values as follows:



**BMI**
UnderweightBelow 18.5Normal18.5–24.9Overweight25.0–29.9Obesity30.0 and Above [60]

The formula is BMI = kg/m^2^ where kg is a person’s weight in kilograms and m^2^ is their height in meters squared [61].

The BMI measurement is very significant. A 2010 study by Kam J and Taylor D [62] showed that patient management difficulty did not increase until the BMI was in the obese or morbidly obese range, especially in cannulation and venipuncture. More so, obesity levels have reached epidemic proportions in many Asian countries and many of them have rates which are not far from that in the USA [63] with the Centers for Disease Control and Prevention (CDC) report in 2015–2016, of the obesity prevalence in US of 39.8% in adults and 18.5% in youth [64]. The highest rate of obesity in Asia is in Thailand and the lowest is in India. While, China was noted with rapidly increasing cases of obesity that occurred in a remarkably short period of time [63].

#### 4.3.2. Skin color/tone

Skin color/tone meter (Fitzpatrick skin type) classification system was first proposed by Thomas B. Fitzpatrick in 1975 based on a person’s skin color and was used as a standard by healthcare professionals and aesthetic practitioners in the assessment of their patients. It ranges from very light complexion to black complexion, with a scale of I to VI skin type. The complete scale is as follows:


**Skin Type**

**Description**
IVery light complexionIILight complexionIIIMedium complexionIVDarker complexionVDark complexionVIBlack complexion [65]

Various studies showed that darker skin color causes difficulty in viewing the vein [7,46,47,49,65]

Moreover, a study showed that a commercial NIR vascular imaging device was reported with limited value in improving success at first attempt of intravenous (IV) cannulation in children with dark skin color [66]. Aside from the skin color, hairy skin was also noted to cause strong reflectance glare that severely impair the vein contrast. It blocks the skin underneath and the subcutaneous veins has a very low contrast ratio resulting to almost visually unnoticeable in the NIR image [50].

#### 4.3.3. Age

Age as a factor to be considered was clearly described by Dhakshayani M and Yacin S., 2015 [47] in their study. In the elderly, vein access was challenging due to the changes in the vein structure as thin and fragile people lose elasticity while pediatrics have smaller peripheral veins with higher content of subcutaneous fat and are usually prone to vasoconstriction. This parameter was also considered in different studies [29,43,46,49].

#### 4.3.4. Site for venipuncture

**Figure 5:**
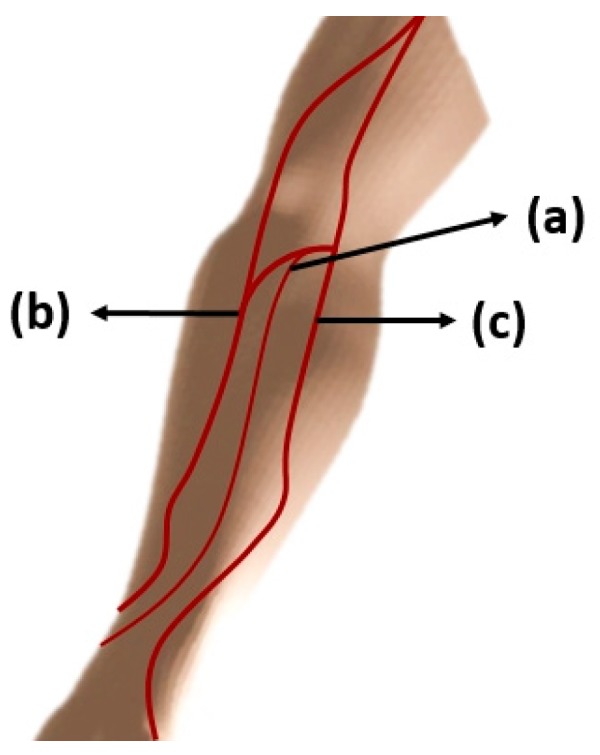
The antecubital fossa with the veins for venipuncture.

The preferred venipuncture sites include the antecubital fossa and the back of the hand that has the superficial vein. Veins are blood vessels that aim to return the blood to the heart. It has a higher percentage of deoxygenated blood (deoxyhemoglobin) as compared to arteries that have higher level of oxygenated blood (oxyhemoglobin) [33]. Hemoglobin (Hb) is the primary component of the blood, specifically; of the red blood cells that have the oxygen carrying capacity [67]. There are three veins that can be selected. Among the three are the veins in the median aspect (center of the arm) must be the first choice followed by the veins in the lateral aspect (outer thumb side), i.e., the cephalic vein, and last are the veins in the medial aspect (inner little finger side), i.e., the basilic vein [68]. Figure 5 shows the main veins of the antecubital fossa (a) median cubital vein, (b) cephalic vein, and (c) the basilic vein. The median cubital vein is considered as the best site for venipuncture. It lies over the cubital fossa and serves as a branching between the cephalic and basilic veins [21]. Compared to the two other sites it is well anchored vein, usually large and prominent [69].

**Figure 6:**
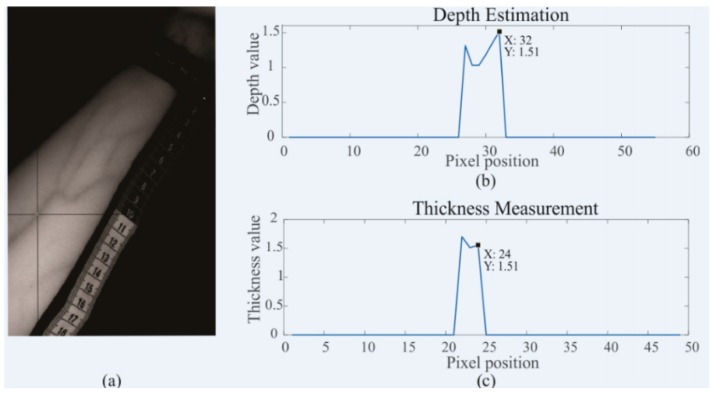
(**a**) Selecting a point from fused images to extract values of: (**b**) Depth and (**c**) thickness using the proposed imaging system [71].

Moreover, there were studies that include the determination of the veins’ properties such as the depth and its diameter as a parameter, which affects its visibility with the use of a vein finder device. The said two parameters were quantitatively measured by the ultrasound method. In the study of Ganesh, S., the ultrasound images were obtained using techniques, such as dot and tick-mark methods, and were analyzed with the software Corel Draw Graphics Suite 12. The diameter of the veins was an average value of the diameter measured in the horizontal and vertical directions reported in (mm) millimeters. This study stated that there is a linear correlation between depth and diameter of veins. [70] Another study by Goh, C. et al. in 2017, which proposed a new measurement system that, infers both the depth and thickness of subcutaneous veins to improve the success rate of venous access. The principle is based on the diffuse reflectance images at three isosbestic wavelengths to measure both the depth and thickness of subcutaneous veins. Measurements were based on the Monte Carlo (MC) method and accomplished by referring an optical density (OD) ratio to a multi-layer diffuse reflectance model. The results of their study were all validated using comparative ultrasound measurements. It shows that the inference of depth and thickness by OD ratio determination, the melanosome (Cm) have to be calculated. A ‘characteristic angle’, as obtained during this process, was then determined and used to select the appropriate Cm group for the determination of the vein depth and thickness. Segmented vein imaging is utilized to specify the location of veins and extract the OD ratio values. The obtained OD ratio is referred to the 3000 diffuse reflectance models to produce depth and thickness, as shown in Figure 6 [71].

The said determinations of the veins depth and its diameter can be considered as a useful parameter for future studies. It can help to improve the capabilities of a vein finder, as a guiding device for cannulation or phlebotomy, performed by medical practitioners.

### 4.4. Assessment of the Vein Finder Prototype

There were 9 out of 21 (42.85%) studies which did not perform human testing with their prototypes that lead to the limitation in the actual evaluation process. For studies with human testing, Cuper, S. et al., 2011 [29] has the highest number of subjects with a total of 125 male and female children followed by Chen A et al., 2013 [49] with 101 patients/subjects and Shahzad A et al., 2014 [7] with 80 subjects/patients, the lowest number of subject was with Dai et al., 2013 [35]. In addition, there is a study that used a synthetic plastic vein model with dog’s blood for their prototype testing.

Access to the antecubital vein is a big challenge for doctors in case of obese people, pediatrics, elderly and dark skinned people as stated in the study of Dhakshayani, M. et al., 2015 [47], with this they specifically selected their subjects as to 25 dark skinned people, 25 obese subjects, 25 pediatrics, and 2 elderly people in testing their prototype by viewing the antecubital vein in forearm and cephalic vein of the dorsal hand.

The prototype tested with pediatric patients for venipuncture showed a positive result, the failure rate with 1 out of 45 (2%) compared without the use of NIR vascular imaging system 10 out of 80 (13%) was noted. The device used was not rated negatively in any of the cases of the performed venipuncture by the phlebotomist [29].

In the testing done by Anupongongarch P et al., in 2015 [37] the prototype was evaluated through actual performance of venipuncture. Results show that the use of the vein finder increased the success of the procedure in the first attempt—15/17 (88.23%) with the device compared to 10/17 (58.82%) by standard of care vein access in pale skin/subject. While, the result from color skin/subjects were 8/13 (61.53%) and 6/13 (46.15%) with and without the use of the device, respectively.

Moreover, the study of Juric S and Zalik B, 2014 [58] included the participation of 43 trainee clinicians (nursing students) in performing the procedure to evaluate their prototype through a non-invasive, observational study having the highest result of 4.45 (mean value) for the Likert scale item (A) “the prototype is useful” and a 35.2% more successful in visualizing and locating veins (n = 500 attempts) than the nursing students.

It is worth mentioning that regarding the device with 15–20 cm distance above the site: The allowable distance was set to ensure the needed space in performing a vein puncture in Kimori et al., 2015 [46] and the prototype by Meng, G. et al., 2015 [48] with a head wearable model. While, there was direct skin contact noted in some of the developed prototypes, the vein finder was introduced with the same purpose as a tourniquet, which is used to help locate the vein. In medical practice, a vein finder is used as a device for venipuncture from one patient to another. With those models that require direct skin contact, it may cause transmission of infection known as hospital acquired infection (HAI) if not properly disinfected. In the 2006 study by Leitch A et al., [72] it was showed that the rate of contamination with Methicillin-resistant Staphylococcus aureus (MRSA) was 32 of 131 (25%) of the tourniquets tested, which leads to the conclusion that the tourniquets being used for phlebotomy may be a potential vectors for transferring bacteria including MRSA. Recently, the Clinical & Laboratory Standards Institute (CLSI) recommended a single-use of tourniquets as a preventive measure in the spread of Methicillin-resistant Staphylococcus aureus (MRSA) and other pathogens, since it is being used with direct contact with patients and might have been contaminated [68,73]. With the vein finder device, a disposable type is not feasible in the current situation; instead a non-contact model can address the concern on how to prevent possible transfer of contamination.

## 5. Conclusions

Based on the review, the development of a low-cost near infrared (NIR) vein finder remains in the phase of improvement, specifically, targeting its aim to lessen the cases of missed peripheral subcutaneous veins during blood collection and intravenous insertion for medication. For the parameter and materials in developing the vein finder prototype, such as optimal wavelength, camera, and sensors and the other materials used by different researchers; they were noted to be still in need of further evaluation. With regards to the assessment process, since it is being challenged by different human factors, increasing the number of parameters and participants/human for actual testing of the prototypes must also be taken into consideration for possible commercialization. Moreover, thorough training for individuals performing cannulation assisted with the vein finder will also help to resolve concerns. Finally, it was noted that publications regarding the assessment of phlebotomists and other medical practitioners with their performance in cannulation using vein finders were limited, specifically showing the success of needle insertion into the vein on the first trial.

## Figures and Tables

**Table 1:** Statistical report on pre-analytical errors (2010–2017).

STUDY (First Author, Year of Publication)	PRE-ANALYTICAL ERROR (%)
West J et al., 2017 [12]	up to 68.20
Najat D, 2017 [13]	up to 70.00
Salinas M et al., 2015 [14]	60.00 to 70.00
Patra S, 2013 [15]	46.00 to 68.20
Hammerling J, 2012 [16]	46.00 to 68.20
Lippi G et al., 2011 [17]	60.00 to 70.00
Goswami B et al., 2010 [18]	77.10

**Table 2:** Characteristics of the different prototypes.

Prototype Study First Author (Year)	Light Source Wave Length (nm)	Camera, Sensor and Filters	Considered Parameters and Site for Evaluation	Type of Evaluation *(Human Testing)*
Ayoub, Y. et al. (2018) [33]	850 and 940	Nikon D810 camera (resolution 36.6 MP), Zomei 720 IR filter	Body Temperature, Site: arm	10 subjects
Carlsen, R. et al. (2018) [42]	850	8 MP NoIR Camera (8 megapixel image sensor), Plastic sheet inside floppy disks or negative films, with diffusers, such as tissue paper and frosted window films.	NA	NA—No actual testing done
Chandra, F. et al. (2017) [43]	600–696	NA	(*) BMI, age, and skin color	Tested to 10 patients
Fernandez, R. et al. (2017) [44]	940	GoldEye P-032 SWIR camera (AlliedVision, Stradtroda, Germany), a Swiss Ranger SR-400011 TOF 3D camera (MesaImaging, Zürich, Switzerland)	NA	NA—No patient testing done
Kim, D. et al. (2017) [45]	850	NIR CCD camera (Grasshopper3 GS3-U3-41C6NIR-C, Point Grey Inc., Richmond, BC, Canada) and a high-resolution lens (GMTHR48014MCN, Goyo Optical Inc., Asaka, Japan) 850 nm band-pass filter (BP850-S44.5, Midwest Optical System Inc., Palatine, IL, USA)	(*) NA	NA—No patient testing done
Anupongongarch et al. (2015) [37]	700–1000	NA	Patient’s skin color	17 pale skin, 13 color skin
Kimori, K. et al. (2015) [46]	850	Compact IR-sensitive charged-coupled device (CIS) CCD	BMI, age, hemoglobin, skin color Sites: Cubital fossa and forearm	72 patients
Dhakshayani, M. et al. (2015) [47]	Multispectral imaging IR 740, 765, 770, 780	Web camera with CMOS sensors, IR pass filters—Kodak wratten 87 IR filter infrared (IR) photographic film	Age, body mass, skin color Sites: antecubital vein, cephalic vein, forearm, and dorsal hand	25 dark skinned people, 25 obese subjects, 25 paediatrics, and 2 elderly
Meng, G. et al. (2015) [48]	830 and 850	Vuzix STAR 1200XL eyewear system IR CCD camera	NA	NA—No patient testing done
Marathe, M. et al. (2014) [31]	920	CMOS camera, captured image is compressed in Joint Photographic Experts Group (JPEG) format, IR filter	NA Site: wrist	NA—No patient testing done
Juric, S. et al. (2014) [24]	740	Standard (Universal Serial Bus) USB camera Pass-through filter (exposed and developed empty 35 mm camera film	BMI	72 subjects
Shahzad, A. et al. (2014) [7]	800–850	Spectral Camera PS V10E	Skin tone: Fair, light brown, dark brown, and dark	80 subjects/patients
Chen, A. et al. (2013) [49]	940	Two monochrome FireWire cameras with high sensitivity CCD sensors in the near-infrared range (Point Grey Firefly MV) 850–1060 nm band pass filters (Edmund Optics)	Age, gender, BMI and skin color (Fitzpatrick skin type)	101 patients
Dai et al. (2013) [35]	850	Monochrome NIR CMOS camera (EO-0413BL Edmund) with DLP projector (DL3000 Texas Instruments)	Site: Hand	(1) Hand vein
Lee, S. et al. (2013) [41]	740	Real-time camera IR long-pass filter 695 nm	(*) NA	Vein model (plastic tube and dog’s blood)
Wang, F. et al. (2013) [50]	Multispectral imaging IR 850, 615, 570, 546, 475	Spectrocam™ Multispectral Imaging Camera (Ocean Thin Films, Golden, Colorado) NIR enhanced CCD camera (a Sony ICX285 sensor) through a Carl Zeiss Distagon 2.8/25 mm ZF-IR lens With multi filters	Asian male, Caucasian male, skin tone, hairy forearm	3 human subjects
Jin et al. (2012) [51]	940	Camera with CMOS high-transmittance imaging lens Filter passing wavelength of 940 nm	Sites: Hand and arm	(1) Hand and arm
Chakravorty, T. et al. (2011) [32]	850	Webcam with OV9650 sensor Liquid Crystal Display (LCD) screen ARM9 single board computer	Site: Finger	NA—No patient testing done
Cuper, N. et al. (2011) [29]	850	IR-sensitive camera with Video Graphics Array (VGA) resolution (640 × 480) Filter blocking all light less than 800 nm.	Children (0-6 years) male and female Dark skin, fat padding,	Children tested: 80 without NIR light, 45 with the NIR prototype
Nundy, K. et al. (2010) [52]	740–760	Ordinary camera phones with even VGA quality pictures Optical filter using butter paper and filter made from exposed and developed film strips	NA	NA—No patient testing done
Crisan, S. et al. (2007) [53]	740–760	Camera with CCD Polarizing filters and blank sheet made of polycarbonate	NA	NA—No patient testing done

Legend: NA—Non applicable, BMI—Body Mass Index, SWIR TOF—short-wave-infrared camera, Time-of-flight camera, CCD—Charge-coupled device, CMOS—Complementary metal oxide semiconductor, *—Requires direct skin contact.
